# A deep learning framework for noninvasive fetal ECG signal extraction

**DOI:** 10.3389/fphys.2024.1329313

**Published:** 2024-04-22

**Authors:** Maisam Wahbah, M. Sami Zitouni, Raghad Al Sakaji, Kiyoe Funamoto, Namareq Widatalla, Anita Krishnan, Yoshitaka Kimura, Ahsan H. Khandoker

**Affiliations:** ^1^ College of Engineering and Information Technology, University of Dubai, Dubai, United Arab Emirates; ^2^ Department of Biomedical Engineering, Khalifa University, Abu Dhabi, United Arab Emirates; ^3^ Department of Industrial and Systems Engineering, Khalifa University, Abu Dhabi, United Arab Emirates; ^4^ Tohoku University School of Medicine, Sendai, Japan; ^5^ Health Engineering Innovation Center (HEIC), Department of Biomedical Engineering, Khalifa University, Abu Dhabi, United Arab Emirates; ^6^ Children’s National Hospital, Washington, DC, United States

**Keywords:** biomedical signal processing algorithms, deep learning, fetal heart rate, long short-term memory, noninvasive fetal electrocardiogram

## Abstract

**Introduction:** The availability of proactive techniques for health monitoring is essential to reducing fetal mortality and avoiding complications in fetal wellbeing. In harsh circumstances such as pandemics, earthquakes, and low-resource settings, the incompetence of many healthcare systems worldwide in providing essential services, especially for pregnant women, is critical. Being able to continuously monitor the fetus in hospitals and homes in a direct and fast manner is very important in such conditions.

**Methods:** Monitoring the health of the baby can potentially be accomplished through the computation of vital bio-signal measures using a clear fetal electrocardiogram (ECG) signal. The aim of this study is to develop a framework to detect and identify the R-peaks of the fetal ECG directly from a 12 channel abdominal composite signal. Thus, signals were recorded noninvasively from 70 pregnant (healthy and with health conditions) women with no records of fetal abnormalities. The proposed model employs a recurrent neural network architecture to robustly detect the fetal ECG R-peaks.

**Results:** To test the proposed framework, we performed both subject-dependent (5-fold cross-validation) and independent (leave-one-subject-out) tests. The proposed framework achieved average accuracy values of 94.2% and 88.8%, respectively. More specifically, the leave-one-subject-out test accuracy was 86.7% during the challenging period of vernix caseosa layer formation. Furthermore, we computed the fetal heart rate from the detected R-peaks, and the demonstrated results highlight the robustness of the proposed framework.

**Discussion:** This work has the potential to cater to the critical industry of maternal and fetal healthcare as well as advance related applications.

## 1 Introduction

The ever-increasing interest in artificial intelligence, machine learning, and Internet of Things (IoT) has yielded significant advancements in various domains and applications (Dhombres et al., 2022). More specifically, the remarkable benefits of employing intelligent machines in the healthcare sector have attracted the attention of healthcare providers and decision-makers to invest in such applications (Signorini et al., 2020; Liu et al., 2020). This resulted in numerous studies that focused on providing techniques to better understand the current health status of patients (Odendaal et al., 2019; Diab et al., 2021). Such studies that focus on providing techniques for monitoring the health status of the heart include, but are not limited to, support vector machines to aid the auscultation procedure using computed tomography scan images () machine learning model based on activity tracker data to classify patient health status (Meng et al., 2020), a bidirectional long short-term memory (bi-LSTM) regression network for noninvasive heart rate (HR) estimation from ballistocardiogram signals (Jiao et al., 2021), a binary classification model for assessing the neonatal heart and lung sound quality for the heart, and breathing rate estimation for telehealth applications (Grooby et al., 2021).

The process of recording the electrical activity of the heart is accomplished noninvasively through electrocardiography. The produced signal, called the electrocardiogram (ECG), graphs the voltage *versus* time of the heart’s electrical activity, showing heartbeats as impulses. Any change in the ECG signal can flag a potential issue with the health of the heart (Melillo et al., 2013).

Fetal ECG monitoring in the early stages of pregnancy is vital for detecting potential health issues related to the fetus (Wahbah et al., 2021; Vo et al., 2020). This will eventually lead to lowering morbidity rates and saving costs Deng et al., (2023). This research is proposed in conjunction with the situation imposed by harsh circumstances such as earthquakes, pandemics (Wu et al., 2020), war conditions, and low-resource settings. In such situations, many cities suffer from a lack of feasibility in accessing hospitals and health clinics worldwide. In addition, a lot of healthcare facilities experienced a huge demand for immediate healthcare attention for people affected by the harsh situation. This resulted in reduced attention to other types of patients, such as pregnant women. Regular and important health checkups on fetal health and development, which are needed more frequently in the last month of pregnancy, could not have been feasible unless the pregnant mother visited the clinics in person. This calls for immediate technological advancements that enable more convenient monitoring of fetal and maternal health.

Monitoring fetal wellbeing has long been performed using Doppler ultrasound technology (Marzbanrad et al., 2018). This method, however, requires good clinical practice administered by highly skilled technicians, which might not be feasible in low- and middle-income settings. In addition, the accuracy of assessing fetal development using this method, which measures the crown–rump length of the fetus, decreases beyond the first trimester of pregnancy (The American College of Obstetricians and Gynecologists, 2017). Another monitoring technique for fetal health involves measuring the ECG signal from the fetal scalp (Bakker et al., 2004). Nevertheless, this invasive approach is risky, as it can lead to infection (Leeuwen et al., 2014). On the contrary, fetal ECG monitoring through the noninvasive recording of the maternal abdominal signal is a promising technique that mitigates the aforementioned limitations (Wahbah et al., 2021; Khandoker et al., 2020).

In this article, we investigate how fetal development can be monitored through noninvasive ECG technology, as accurate assessment is imperative for optimal maternal and neonatal outcomes. To achieve this, a deep learning architecture based on long short-term memory (LSTM) is utilized. Such networks showed promising results in works focusing on fetal health, including predicting estimated fetal weight (Naimi et al., 2018), identifying predictors of fetal growth abnormalities (Kuhle et al., 2018), and predicting fetal gestational age and neurodevelopmental maturation based on 3D ultrasound brain images (Namburete et al., 2015).

On the other hand, the state-of-the-art lacks research works that focus on analyzing the fetal HR, which can be estimated using automatically extracted R-peaks of the fetal ECG signal through a deep learning model. Therefore, we took one further vital step by computing the fetal HR from the detected fetal R-peaks and enhancing it by introducing the fetal heart rate enhancement (FHRE) technique. This is vital for assessing the development of the fetus and fundamental to neurological screening, which is essential for reducing fetal deaths.

In this article, a novel approach is developed to extract and identify the R-peaks of the fetal ECG signal using noninvasively recorded signals from the abdominal area of the mother, known as the abdominal signal. This signal combines both the maternal and fetal ECGs, in addition to noise. The dataset used in this study includes ECG signals collected from 70 pregnant women with no records of fetal abnormalities. Patients’ data have been obtained from the Children’s National Hospital in the United States of America as well as from Tohoku University Hospital and Kanagawa Children’s Medical Center in Japan. The proposed methodology is based on a deep learning architecture, which has efficient capabilities for applications related to fetal cardiovascular signals (Garcia-Canadilla et al., 2020). After extracting the fetal ECG, we implement a post-processing technique that we refer to as fetal ECG post-processing (FECGPP). The presented framework is fully automated and can be easily utilized by non-experts with little training and limited resources, which is ideal for low- and middle-income settings.

The major contributions of the work presented in this article include the following:

1) Introducing the fetal R-peaks labeling (FRPL) method to robustly label the R-peaks from the acquired abdominal signals.
2) Efficiently detecting the fetal ECG signal and extracting it from the abdominal composite signal by employing the introduced deep learning model.
3) Improving the extracted fetal ECG by applying the FECGPP technique.
4) Estimating the fetal HR from the detected R-peaks of the ECG and implementing a post-process technique (FHRE) to enhance the estimated fetal HR.

The remainder of the article is organized as follows: Section 2 discusses the FRPL method and the deep learning architecture that are proposed in this article for efficiently extracting the fetal ECG signal from the abdominal composite signal, along with the implemented algorithm to post-process the fetal HR. The results of this study, including the leave-one-out cross-validation, correlation and agreement analyses, and fetal HR computation, are shown in Section 3 and discussed in Section 4. Lastly, we present the conclusion in Section 5.

## 2 Materials and methods

In this section, the dataset used in this study to train and test the network is discussed and explained. Additionally, the proposed FRPL method is illustrated, along with the deep learning model utilized for fetal R-peak detection from the composite abdominal signal. The cutoff for significance used while conducting the statistical analysis in this article is set at *P* < 0.05.

### 2.1 Dataset

The dataset of abdominal ECG signals from 70 pregnant women with healthy fetal conditions was obtained from Children’s National Hospital in the United States of America (12 samples, 17%), in addition to Kanagawa Children’s Medical Center (13 samples, 19%) and Tohoku University Hospital (45 samples, 64%) in Japan. The study protocols were approved by the Tohoku University Institutional Review Board (IRB: 2020-1–951 and 2015-2–80–1) and the Children’s National Hospital IRB with appropriate institutional agreements. Written informed consent was obtained from all subjects. All experiments were performed in accordance with relevant guidelines and regulations.

Abdominal signals were recorded bipolarly from the 12 electrodes placed on the maternal abdomen. The sampling frequency was 1 kHz, and the resolution was 16 bits. ECG signals were recorded with the participant in the supine position, and only part (1-min length) of the recorded period was used in this work. Moreover, the data were subjected to filtration using a bandpass filer with a bandwidth of 0.05–100 Hz, and a notch filter afterward was used to remove 50 Hz or 60 Hz noise due to the ECG data acquisition device.

### 2.2 Fetal R-peak labeling method

**FIGURE 1:**
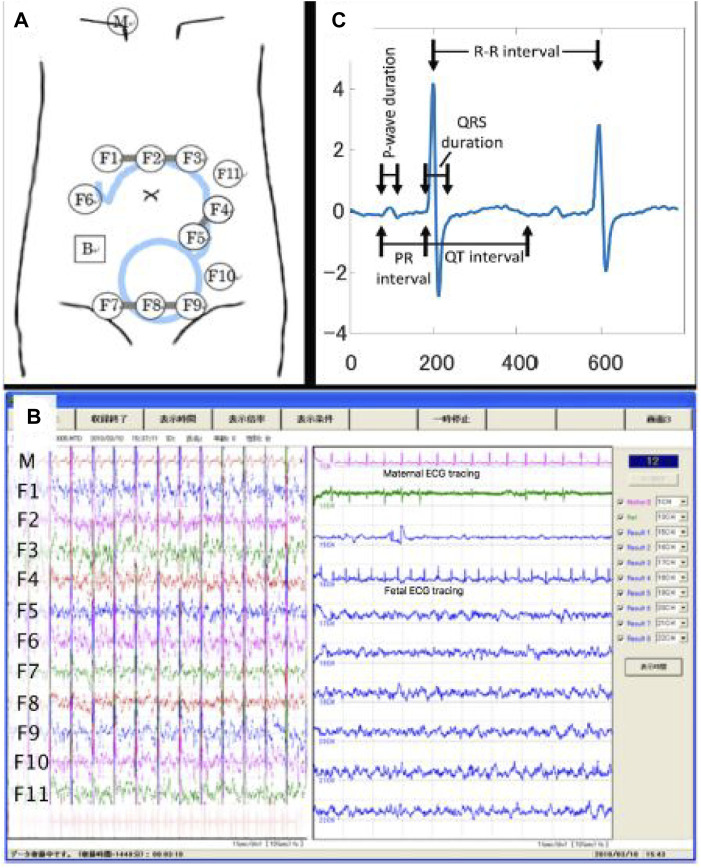
Configuration of data acquisition for the raw (i.e., original) abdominal signal. **(A)** Experimental setup illustrating the scheme describing the position of the 12 electrodes on the maternal abdomen. **(B)** The real-time display while performing data acquisition. **(C)** Representative signal-averaged fetal ECG tracing with measured intervals. Details regarding the experimental setup could be found in our previous study (Doshi et al., 2019). Adapted from Doshi et al., 2019, with permission from SNCSC.

Initially, the fetal ECG signal was separated from the abdominal composite signal by employing the method reported by Sato et al. (2007), which combines cancellation of the maternal ECG signal and blind source separation with a reference (BSSR). The experimental setup is described in detail in our previous study by Doshi et al. (2019), and Figure 1 shows the experimental setup by illustrating a scheme describing the position of the 12 electrodes on the maternal abdomen, in addition to real-time display while performing data acquisition, along with representative signal-averaged fetal ECG tracing with measured intervals.

Moreover, the location of the R-peaks of the fetal ECG signal was detected by a custom-made MATLAB (version R2022b) routine program similar to our previous work by Wahbah et al. (2021).

**FIGURE 2:**
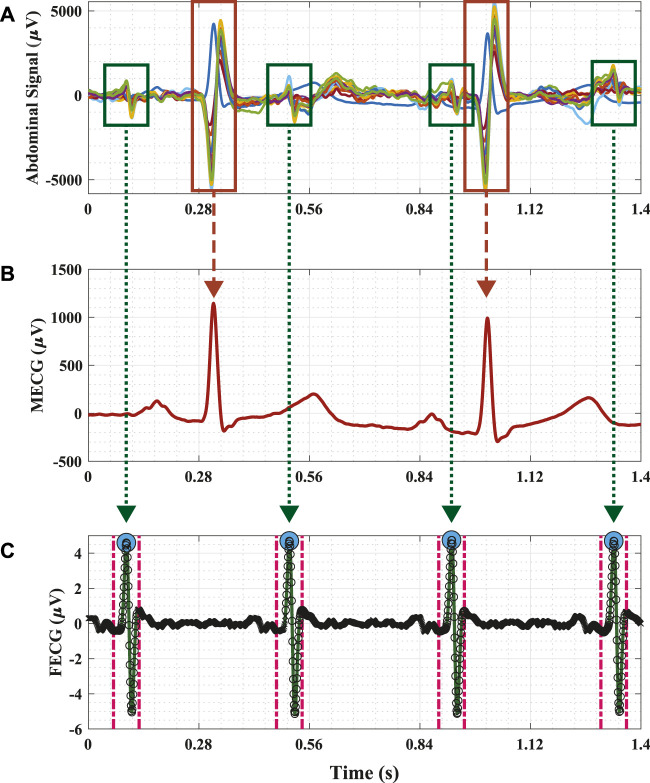
Example of the process for obtaining the ground truth for the proposed deep learning framework for identifying the fetal R-peaks in the electrocardiogram signal. **(A)** 12-channel raw (i.e., original) abdominal time-series signal that is a composite of the maternal ECG, fetal ECG, and noise. The red and green boxes identify the maternal and fetal ECGs, respectively, within the abdominal signal. **(B)** The maternal ECG time-series signal is solely recorded by one of the electrodes (i.e., channels). **(C)** The fetal ECG time-series signal was extracted using the BSSR method (Sato et al., 2007) and is considered the ground truth in this study. The blue-filled circle markers (i.e., 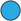) illustrate the exact location of the fetal R-peak in the ECG signal. The proposed FRPL method is depicted in this panel by labeling each of the displayed time segments using a cross marker (i.e., ×) to annotate Class 0 and a hollow circle marker (i.e., O) to annotate Class 1. The time period at which each R-peak of the fetal ECG appears is assigned by the proposed method as 65 ms and is demonstrated as dashed magenta vertical lines. *μ*, micro; ECG, electrocardiography; s, seconds.

Second, we measured the time duration for the fetal QRS—to be considered a true positive for a sampling frequency of 1 kHz—as approximately 70 ms . For the same sampling frequency, the work introduced by Vo et al. (2020) reported this duration to be 50 m. In this study, we set the segment duration as 65 ms (i.e., within the range of the two previous values) to identify the fetal QRS. Therefore, input window frames (65 m duration each) are labeled as Class 1 if the fetal R-peak annotation exists within. To depict this, an example of the process for obtaining the ground truth for the proposed deep learning framework for extracting the fetal ECG signal is demonstrated in Figure 2. The raw (i.e., original) abdominal time-series signal that was recorded using the 12-channel electrodes along with the maternal ECG signal are plotted in Panels (a) and (b), respectively. Moreover, the figure also displays the fetal ECG from the BSSR method (Sato et al., 2007), which is considered the ground truth in this study in Panel (c). This signal is used to demonstrate the proposed FRPL method in operation by overlaying the original labels that we assigned in reference to the ground truth signal and that indicate the period at which the R-peaks of the fetal ECG appear (i.e. 65 m). The different elements in the figure are displayed using multiple colors, line styles, and markers. Three types of annotations are used in the scatterplot, including a blue-filled circle marker (i.e., 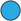), which illustrates the exact location of the fetal R-peak in the ECG signal; a cross marker (i.e., ×), which refers to Class 0 (i.e., the location of the fetal R-peak does not exist within the 65 ms); and a hollow circle marker (i.e., O), which indicates Class 1 (i.e., the fetal R-peak appears within the determined time segment). Furthermore, the assigned time period of 65 ms at which each R-peak of the fetal ECG appears is demonstrated as dashed magenta vertical lines. To summarize, the fetal ECG signal was divided into segments of 65 ms, which are marked with Class 1 label if the R-peak reference annotation appears within; otherwise, Class 0 label is used.

### 2.3 Training and testing scheme

The generated labels (i.e., Classes 0 and 1) on the previous segments (duration of 65 ms each) of the 1-min fetal ECG are used in training the proposed deep learning model. Initially, the original 12-channel abdominal signal (1 min duration) is divided into segments based on the fetal input frames (65 ms duration). The generated labels are used afterward in the proposed model to make the algorithm detect the fetal ECG signal. Therefore, the model identifies the fetal ECG among the other composite signals (i.e., among the remaining inputs from 11 channels).

Data standardization is applied to training and testing datasets using the training set mean and standard deviation (SD). It is worthwhile noting that the training dataset comprises 75% of the overall dataset and the testing set accounts for the remaining 25%. Demographics for the dataset are listed in Table 3.

Data standardization has been applied to the signals as follows:

$$z=\frac{X-\mu }{\sigma },$$ (1)

where *z* is the standardized value (i.e., a z-score), *X* is the observed value, *μ* is the mean, and *σ* is the standard deviation. Model cross-validation has been accomplished through two standard tests: subject-dependent (5 folds) and subject-independent (leave-one-subject-out).

### 2.4 Fetal ECG detection network

**FIGURE 3:**
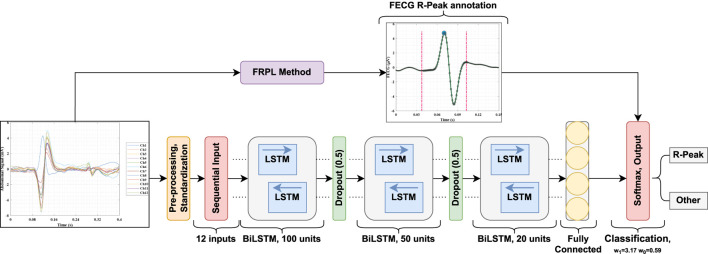
A block diagram of the proposed fetal ECG detection framework incorporating the FRPL method.

In this section, the implemented deep learning network that is based on the LSTM architecture is introduced and illustrated. Figure 3 displays a block diagram of the proposed fetal ECG R-peak detection framework to enhance the reader’s understanding while depicting the extraction strategy adopted in this work. Furthermore, the configuration of the proposed architecture, including the layers’ types, number of activations, and parameters, is shown in Table 1. The used network mainly consists of three bidirectional LSTM (BiLSTM) layers, with 100, 50, and 20 hidden units, respectively. We chose to use an LSTM-based network since it has been proven in the literature to be efficient for the classification of ECG data (Zhou et al. (2021); Zitouni and Khandoker, 2022). Additionally, two dropout layers were used between the BiLSTM layers, as well as a fully connected and a softmax classification layer. The selected number of hidden units in the BiLSTM layers was based on the number of samples in the used dataset, and the use of dropout layers helps prevent over-fitting. The abdominal ECG data are first pre-processed, standardized, and then segmented into 65 m segments, as described previously. Each segment is inputted into a sequential input layer with 12 feature inputs corresponding to the 12 abdominal ECG channels. Additionally, the proposed FRPL method is applied to the data to automatically annotate the segments of data to R-peaks or others for the training and validation processes. Weighted classification was implemented, which helps rectify the effect of the imbalance in data since the number of data segments that contain R-peaks is less than that of the other segments. Each class’s weight is computed as follows:

$$w_{c}=\frac{ns}{\left(nc\times ns_{c}\right)},$$ (2)

where *w*_*c*_ is the weight of each class, *ns* is the total number of samples, *nc* is the number of unique classes, and *ns*_*C*_ is the number of samples in the respective class. Thus, the weight for the R-peak class was *w*_1_ = 3.17, while the weight for the other class was *w*_0_ = 0.59. Finally, R-peaks are detected by distinguishing segments containing them from others.

**TABLE 1:** Parameter settings of the proposed detection architecture.

Layer	Activation	Parameter
Sequence input	12	–
BiLSTM	400	Input: 1600 × 12; recurrent: 1600 × 200
Dropout (0.5)	400	–
BiLSTM	200	Input: 800 × 400; recurrent: 800 × 100
Dropout (0.5)	200	–
BiLSTM	100	Input: 400 × 200; recurrent: 400 × 50
Fully connected	2	Input: 2 × 100
Softmax	2	–
Classification	2	–

### 2.5 Fetal ECG post-processing

**FIGURE 4:**
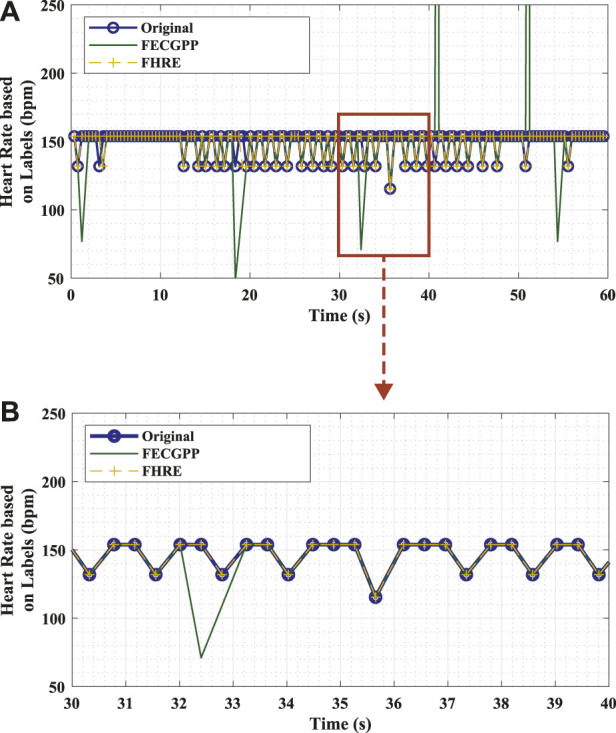
Example of fetal HR time-series signals that were computed from the R–R intervals based on original labels (ground truth, blue) and estimated labels [deep learning algorithm: proposed ECG post-processing (FECGPP, green) with fetal HR enhancement (FHRE, yellow)]. **(A)** Visual representation of the desired output illustrated using a section of the time-series signal displayed for 60 s. **(B)** Zoomed-in section of the signal displayed for 10 s. Bpm, beats per minute; s, seconds.

Figure 4 visually shows the desired output and highlights the significance of the proposed deep learning framework in removing the outliers so that the output signal aligns with the ground truth. To elaborate, the figure shows an example of the fetal HR time-series signals that correspond to different labels (original in comparison with the deep learning framework) of the fetal R-peaks from the ECG signal that was recorded for 1 min. The HR signal, in beats per minute (bpm), is computed as the difference in locations between consecutive R-peaks (i.e., R–R interval) in a specific time period and is expressed by Li et al. (2007) as

$$Heart\;Rate=\frac{60}{R-R\;Interval}.$$ (3)

In this study, different class labels were expanded for 65 ms in order to obtain the R–R intervals. The fetal HR was computed from consecutive R–peaks for a window length of 10 s, which is common in clinical practice (Li et al., 2007).

**FIGURE 5:**
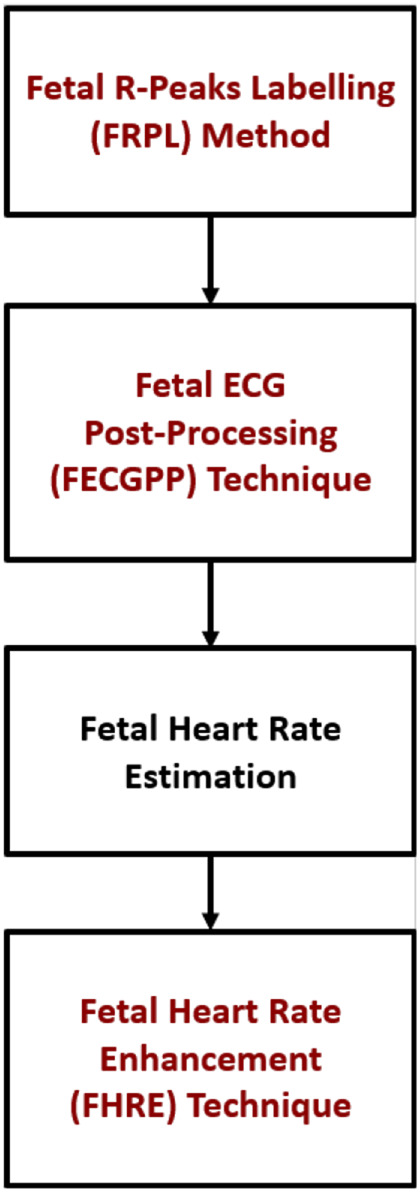
Block diagram showing the main blocks in the proposed framework.

Figure 5 shows the proposed procedure, starting with the fetal R–peak labeling method, the ECG post–processing technique, the computation of fetal HR, and the HR enhancement technique. FECGPP is primarily proposed as a further improvement to the detection results. Particularly, it is aimed at reducing the amount of false detection, which results from data imbalance due to the fact that the number of R–peaks in the ECG signal is much smaller than other parts of the signal.

### 2.6 Fetal heart rate enhancement

The FHRE method is applied to the predicted fetal HR to remove any artifacts by implementing the signal-dependent rank-order mean (SD–ROM) algorithm (Chandra et al., 1998) and adaptive filtering (Wessel et al., 2000). As such, the role of FHRE is merely that of a post-processing technique in which the filter type is not of great significance.

## 3 Results

This section demonstrates the performance of the proposed framework by showcasing the results associated with two categories: the detection performance and improved measures when implementing the deep learning-based framework and the accuracy for fetal HR estimation using the identified fetal R-peaks by the proposed framework.

The first category shows the results for both subject-dependent and -independent validation experiments. The experiments were conducted using MATLAB (Version R2022b), where the Deep Learning Toolbox was utilized to design the detection network. During the training phase of the experiments, we aimed to strike a balance between the number of epochs and the minimum sequence length to ensure a reasonable training speed while still having sufficient iterations for high accuracy. We conducted the tests with the number of epochs ranging from 80 to 100. The minimum sequence length was set to 65, matching the length of the input sequences. We also employed a scheduled learning rate, starting at 0.0005 and dropping by a factor of 0.1 halfway through the epochs. Additionally, the value of the gradient threshold was set to 1. These hyper-parameters were selected after preliminary experimentation and testing of the network, with initial parameters inspired by recommendations and works with similar types of data in the literature.

More importantly, the work in this study did not stop only at the first category (which is the step of reporting the performance of the deep learning network); it was indeed carried further to the second category (demonstrating the robustness of the proposed framework in a practical manner by computing the fetal HR). This initiative is a major contribution of the presented work in this article by itself, as it provides a practical and complete picture of the application of the proposed framework. By computing the fetal HR from the labels associated with the R-peaks of the fetal ECG as well as the correlation and agreement analyses, the significance of the proposed framework is highlighted by visual demonstrations using the Pearson correlation coefficient and the Bland–Altman plots.

### 3.1 Subject-dependent experiment

To provide a reliable evaluation of the model’s performance and reduce the likelihood of over-fitting, a *k*-fold cross-validation test was performed. In this study, a 5-fold cross-validation was conducted on the used dataset. Table 2 shows the experiment results, where an average accuracy of 93.0% and an average F1 score of 0.96 were achieved. Furthermore, the proposed FECGPP technique was utilized, yielding improved results, where average accuracy and average F1 score increased to 94.2% and 0.97, respectively.

**TABLE 2:** Five-fold cross-validation results for subject-dependent experiment.

	Fold 1	Fold 2	Fold 3	Fold 4	Fold 5	Average
						
Accuracy (%)	92.5	93.4	93.5	93.2	92.6	93.0
F1 score	0.96	0.97	0.97	0.96	0.96	0.96

**FIGURE 6:**
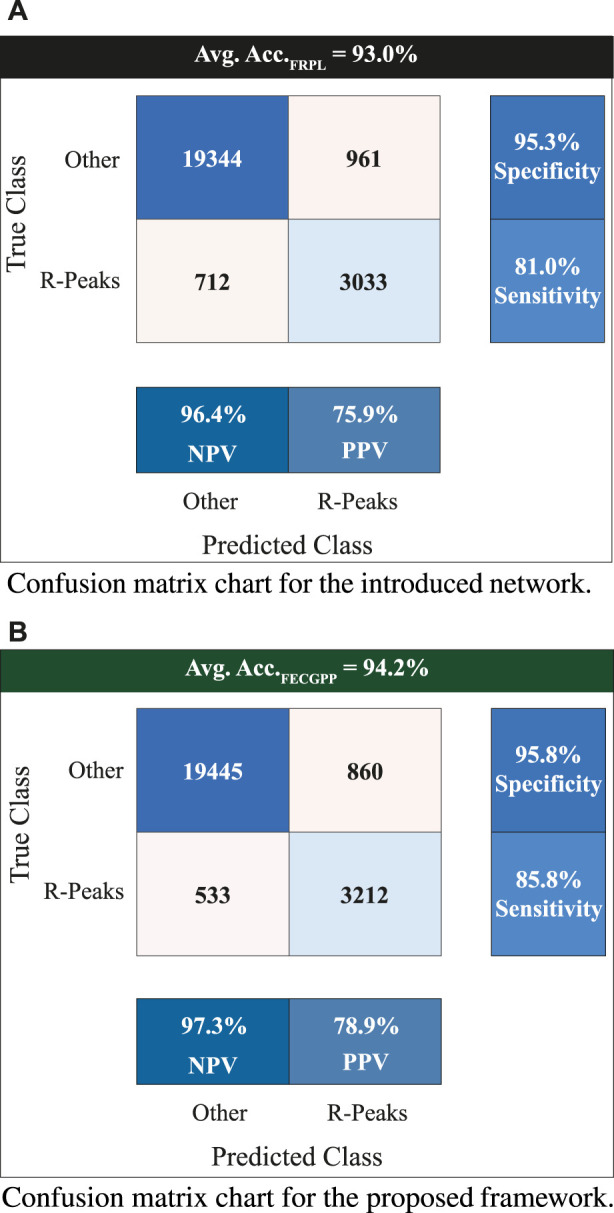
Confusion matrix charts showing the count of true *versus* predicted R-peaks of fetal electrocardiogram. The entries marked with “Other” refer to non R-peak locations in the fetal signal. The displayed value at the top of each panel represents the average accuracy when performing the 5-fold test. The two panels are associated with **(A)** the introduced deep learning network for fetal R-peak identification from the raw (i.e., original) ECG abdominal signal and **(B)** the proposed FRPL method when applied along with the introduced network. All quantitative measures show an improved performance when incorporating the proposed FRPL method in the implemented framework. Avg, average; Acc, accuracy; NPV, negative predicted value; PPV, positive predicted value (i.e., precision).

**FIGURE 7:**
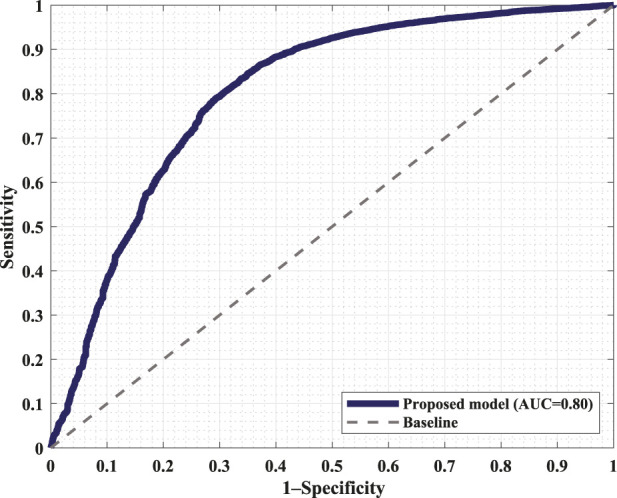
Receiver operating characteristic (ROC) curve of the proposed framework incorporating the FRPL method along with the deep learning network. The panel demonstrates the false peaks vs. true peaks, along with that of a random classifier denoted as the baseline. AUC, area under the curve.

Figure 6 displays the confusion matrix corresponding to the obtained results. The two panels show the detection performance of the deep learning network and the improved measures when the proposed FRPL method is applied. Moreover, the ROC curve of the proposed framework is shown in Figure 7.

**FIGURE 8:**
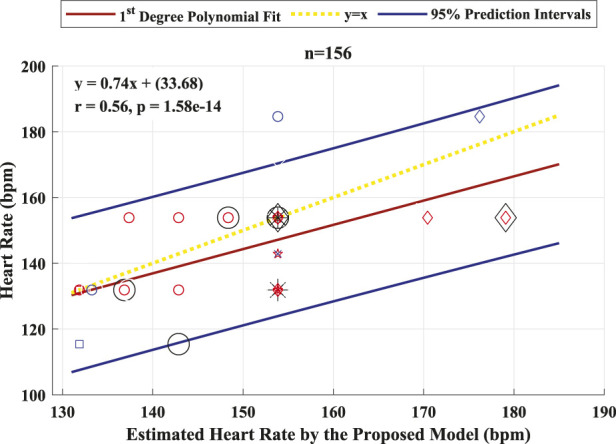
Pearson correlation plot between the fetal HR computed from the R–R intervals that are based on the original labels of R-peaks and predicted values by the proposed algorithm for 26 healthy fetuses. The linear polynomial fit (regression) line along with the 95% prediction intervals are shown as solid red and blue lines, respectively. The identity line is represented as a dotted yellow line. *P* < 0.05 was considered statistically significant. Bpm, beats per minute; n, number of R–R intervals.

**FIGURE 9:**
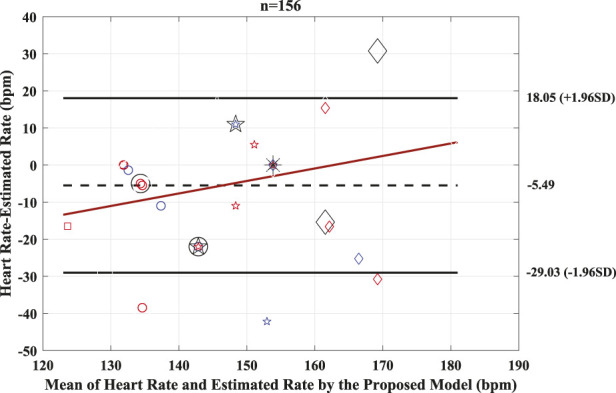
Bland–Altman plot for the predicted and BSSR-based fetal heart rate signal (sample size = 26 subjects). Bias is shown as dashed black line, limits of agreement (±1.96×SD) are shown as solid black lines, and regression fit of the differences on the means is represented as solid red lines. Bpm, beats per minute; n, number of R–R intervals.

The performance of the proposed model to detect and identify the fetal ECG from the composite abdominal signal was validated against the method of BSSR (Sato et al., 2007). Figure 8 (Pearson correlation plot) presents the significant (*P* < 0.05) correlation between the fetal HR computed from the R–R intervals that are based on the original labels of R-peaks and predicted values by the proposed framework with an *r* value of 0.56. Additionally, Figure 9 shows the Bland–Altman plot of the fetal HR comparing the proposed framework with the method of BSSR (i.e., ground truth). The plot validates that estimated HR values are within the limits of agreement (LoA) (i.e., ±1.96 ×SD). The estimated bias (i.e., mean differences) is −5.49 weeks, and the values of the LoA are 18.05 and −29.03 weeks, respectively.

### 3.2 Subject-independent experiment

To further validate the generalizability and applicability of the proposed framework on new unseen data (Zitouni et al., 2023), subject-independent experiments were also performed. The subject-wise leave-one-out cross-validation was performed using 26 subjects. In this experiment, each time training is performed using data from all subjects except the one on whom FECG extraction is applied. Thus, no prior knowledge of the testing subject data exists in the network.

The patients’ demographics along with the test result values when performing the subject-dependent leave-one-out cross-validation are listed in Table 3. This experiment resulted in an average accuracy of 88.8 ± 6.4.

It may be noted that the data of the remaining 44 patients were excluded from this test because their information could not be disclosed under the IRB and thus could only be used for training.

**TABLE 3:** Demographics of the dataset population (N = 26 subjects), along with the subject-dependent leave-one-out cross-validation test accuracy for each patient.

No.	Maternal health status	Gestationalage (weeks)	Fetalweight (g)	Maternalage (years)	Body massindex (kg/m^2^)	Accuracy (%)
1	Normal	20.6	393	33	32.4	93.8
2	Exists (autoimmune disease and protein S hypothyroidism)	39.3	2,835	35	21.0	90.2
3	Normal	20.1	355	31	23.9	88.5
4	Exists (uterine/appendix disease and autoimmune disease)	26.2	937	32	15.8	88.7
5	Exists (uterine/appendix disease and autoimmune disease)	33.4	1,854	28	33.0	68.5
6	Exists (History of hepatitis B)	30.0	1,310	22	24.1	92.9
7	Exists (others [history of hydatidiform mole])	26.3	1,023	24	25.9	92.9
8	Normal	24.0	757	31	24.2	93.8
9	Exists (uterine/appendix disease)	29.2	1,239	35	21.0	89.4
10	Exists (autoimmune disease and first birth in old age)	38.3	2,267	35	21.3	94.4
11	None	35.1	–	36	–	97.0
12	Exists (respiratory disease and uterine and appendage disease)	39.2	2,892	45	35.6	86.9
13	None	39.3	3,138	30	26.2	94.1
14	Normal	35.4	2,462	41	21.7	90.5
15	Normal	25.3	916	38	20.0	84.3
16	None	35.1	2,281	32	21.9	97.1
17	Exists (autoimmune disease)	26.0	919	32	26.9	86.4
18	Exists (uterine/appendix disease and autoimmune disease)	29.0	1,314	34	21.5	94.1
19	Exists (uterine/appendix disease)	24.3	809	29	24.7	77.7
20	Normal	27.2	1,019	22	23.1	86.8
21	Normal	29.2	1,150	22	23.8	84.2
22	Exists (blood disease)	29.4	1,445	41	20.4	86.5
23	Exists (uterine/appendix disease and autoimmune disease)	34.0	2,252	34	22.5	85.0
24	Exists (uterine/appendix disease)	28.5	1,383	29	25.6	90.4
25	Exists (autoimmune disease)	30.6	1,481	33	20.9	95.0
26	Exists (autoimmune disease)	31.6	–	25	20.1	81.0
		30.3 ± 5.6¶	1,518 ± 794¶	31.9 ± 5.9¶	23.9 ± 4.4¶	88.8 ± 6.4¶

Standard deviation (SD).

¶: mean ±SD.

## 4 Discussion

This study successfully demonstrated that the proposed framework could be adopted as an accurate tool to identify and separate the fetal ECG signal from the composite abdominal signal by detecting the fetal QRS peak locations. The recorded ECG signals were considered for a recording length of 1 min per subject. To prepare the dataset, the method of BSSR (Sato et al., 2007) was employed to separate the fetal ECG from the composite abdominal signal. Fetal QRS peak locations were detected afterward by a custom-made MATLAB (version R2022b) routine program and labeled for every segment in the training dataset. This is considered one of the key highlights of this study. The abdominal ECG signals were segmented based on the data labels and inputted into the LSTM-based detection model.

The assessment metrics for evaluating the proposed approach for detecting the fetal R-peaks from the composite abdominal signal revealed robust performance. A 5-fold cross-validation scheme was used to validate the proposed approach, which resulted in an average accuracy of 93.0% when performing the test. Additionally, the computed F1 score is 0.96. The FECGPP technique focuses on improving the detection of the peaks by reducing the false positives and, more importantly, reducing the false negatives and increasing the true positives (detected peaks). Thus, after applying the FECGPP, the accuracy improved to 94.2% and the F1 score to 0.97. More importantly, the sensitivity (i.e., true-positive rates) has increased from 81% to 85.8%. This leads to better overall performance and, hence, a more robust estimation of the fetal HR.

After estimating the fetal HR, it can be depicted from the zoomed section on the lower panel of Figure 4 that the HR signals generated from the ground truth and proposed framework via the FHRE technique are perfectly aligned. When validating the performance of the proposed algorithm, the Bland–Altman plot shows that the higher the R–R intervals, the larger the difference between the evaluated approach and the ground truth. It can thus be speculated that the proposed model seems to work better for shorter R–R intervals. This shows that the method can provide a dependable estimation of the HR value, allowing it to be applicable in the continuous monitoring of fetal health.

When performing the leave-one-subject-out cross-validation (subject-independent test), the proposed framework achieved an average accuracy of 88.8%. The 16 subjects that have healthy maternal conditions and normal BMI values have a prediction accuracy of 90% on average.

Observing the health information of the subject with the lowest accuracy (i.e., Subject#5), the BMI value is outside the normal range, highlighting that it has a unique physique compared to the rest of the dataset. Moreover, this subject has maternal complications, which affect the fetal HR rhythm. In this study, five subjects had a BMI value greater than 26 kg/m^2^. This resulted in a drop in the algorithm’s accuracy to 85% on average. A similar condition can be observed for the three subjects with the lowest accuracy values, making these subjects unique from the overall sample. The results show that the accuracy of patients with a BMI outside the normal range is 84% on average.

The patients who had maternal complications produced an average accuracy of 87%, while those without any maternal conditions had an average accuracy of 91%. These patients are more consistent with each other, resulting in higher accuracy. Considering the subjects without obstetric complications, the average accuracy is 87.5%. This can be speculated to be due to obstetric complications being dependent on the history of previous pregnancies, as opposed to the current one, which has a low effect on the accuracy of the proposed approach.

As for the fetal weight, fetuses with gestational age (GA) younger than 30 weeks had an average accuracy of 88%, while those with GA greater than 34 weeks had an average accuracy of 93%. This strongly indicates that fetal ECG becomes more feasible to extract when the pregnancy is beyond 30 weeks of gestation. This observation is in line with the study reported by van Laar et al. (2014), which mentions that the fetal ECG measurements are extremely difficult to obtain at approximately 30 weeks of gestation due to the presence of the vernix caseosa (Oostendorp et al., 1989; Stinstra and Peters 2002), which electrically shields the fetus from its surroundings. Our fetal ECG peak detection framework, however, achieved an average accuracy of 86.7% even for the challenging period of vernix caseosa layer formation (28–34 weeks of gestation) when performing the subject-independent cross-validation test. Indeed, the results of this study clearly show that the performance of our fetal ECG signal extraction framework increases in proportion to the fetal weight value as gestation progresses. Finally, some sources of errors could be present, such as those due to equipment or humans (i.e., errors while placing the electrodes of the ECG recording device).

This research promotes the development of new technologies and/or enhances existing ones. The conducted research activities addressed some of the barriers associated with noninvasive fetal monitoring in harsh circumstances, pandemics, and low-resource situations. Adopting the proposed approach, which is based on state-of-the-art deep learning networks and the noninvasively recorded abdominal signal, has the potential to cater to the critical industry of maternal and fetal healthcare as well as advance related applications. With this motivation, the proposed framework helps monitor fetal development and can be easily implemented to assist physicians in accurately assessing fetal wellbeing. The application has numerous practical merits. It provides an assistive healthcare tool for physicians and pregnant mothers to monitor the health of the fetus when the ECG signal is recorded. The implementation targets health monitoring in low-resource settings or imposed circumstances that restrict access to the hospital (such as disasters, COVID-19, earthquakes, or similar conditions). The implementation is convenient, can operate anywhere and anytime, and does not require skilled technicians. The key features of the proposed methodology are its low cost, highly improved efficiency value, and easy implementation when any ECG monitoring device is used to record the abdominal signal. In contrast, other maternal and fetal monitoring approaches could have high costs for some patients and could be considered medical devices that may be only accessible by healthcare providers. Such systems could require clinical practice administered by highly skilled technicians, which might not be feasible in low- and middle-income settings.

The proposed work would provide fundamental and transitional research outputs for fetal neurological screening, in addition to its potential to reduce fetal deaths. Being fully automated, the proposed approach can be utilized by non-experts with minimal training and/or limited resources. In addition, it can be implemented to detect health issues related to the fetus in the early stages of pregnancy. This would result in a significant cost and time reduction, as well as mitigate the burden on healthcare providers.

## 5 Conclusion

This article presented a novel approach for extracting and identifying the R-peaks of the fetal ECG signal directly from signals obtained by using 12-channel abdominal electrodes. The proposed framework is based on a combination of the (1) FRPL method for labeling the fetal R-peaks, (2) LSTM-based R-peak detection model, (3) FECGPP for extracted fetal ECG signal improvement, and (4) fetal HR estimation and enhancement through the FHRE technique. The performance of the model is evaluated against the method of blind source separation with a reference, which is used in this study to obtain the ground truth of fetal ECG signals.

The results are presented in two categories: the detection performance and improved measures when implementing the deep learning-based framework and the accuracy for fetal HR estimation using the identified fetal R-peaks by the proposed framework. The first category of results shows that the proposed framework achieved an average accuracy of 94.2% in the subject-dependent test. When performing the subject-wise leave-one-out cross-validation test, the proposed model produced an average accuracy of 88.8%. The best results in this test were for the patients with GA of approximately 35 weeks and no maternal conditions. This revealed that it is more feasible to extract and identify the R-peaks of the ECG signal of fetuses from healthy mothers with advanced age throughout pregnancy. The second category is a major contribution of this work, where it provides a practical and complete picture of the application. When estimating the fetal HR from the labels associated with the identified R-peaks of the fetal ECG, the resulting Pearson correlation coefficient was 0.56, and the Bland–Altman’s bias was −5.49 weeks. These results clearly demonstrate the robustness of the proposed framework for detecting fetal R-peaks from the composite abdominal ECG signal and accurately estimating the fetal HR.

## Data Availability

The datasets presented in this article are not readily available because the data that support the findings of this study are confidential under the Tohoku University Institutional Review Board (IRB: 2020-1-951 and 2015-2-80-1) and Children’s National Hospital IRB with appropriate institutional agreements. Requests to access the datasets should be directed to Prof. Ahsan. H. Khandoker, ahsan.khandoker@ku.ac.ae.
